# Identification of Novel QTLs for Isolate-Specific Partial Resistance to *Plasmodiophora brassicae* in *Brassica rapa*

**DOI:** 10.1371/journal.pone.0085307

**Published:** 2013-12-20

**Authors:** Jingjing Chen, Jing Jing, Zhongxiang Zhan, Teng Zhang, Chunyu Zhang, Zhongyun Piao

**Affiliations:** 1 College of Horticulture, Shenyang Agricultural University, Shenyang, China; 2 National Key Laboratory of Crop Genetic Improvement and College of Plant Science and Technology, Huazhong Agricultural University, Wuhan, China; Nazarbayev University, Kazakhstan

## Abstract

*Plasmodiophora brassicae*, the causal agent of clubroot disease of the *Brassica* crops, is widespread in the world. Quantitative trait loci (QTLs) for partial resistance to 4 different isolates of *P. brassicae* (Pb2, Pb4, Pb7, and Pb10) were investigated using a BC_1_F_1_ population from a cross between two subspecies of *Brassica rapa*, i.e. Chinese cabbage inbred line C59-1 as a susceptible recurrent parent and turnip inbred line ECD04 as a resistant donor parent. The BC_1_F_2_ families were assessed for resistance under controlled conditions. A linkage map constructed with simple sequence repeats (SSR), unigene-derived microsatellite (UGMS) markers, and specific markers linked to published clubroot resistance (CR) genes of *B. rapa* was used to perform QTL mapping. A total of 6 QTLs residing in 5 *CR* QTL regions of the *B. rapa* chromosomes A01, A03, and A08 were identified to account for 12.2 to 35.2% of the phenotypic variance. Two QTL regions were found to be novel except for 3 QTLs in the respective regions of previously identified *Crr1*, *Crr2*, and *Crr3*. QTL mapping results indicated that 1 QTL region was common for partial resistance to the 2 isolates of Pb2 and Pb7, whereas the others were specific for each isolate. Additionally, synteny analysis between *B. rapa* and *Arabidopsis thaliana* revealed that all *CR* QTL regions were aligned to a single conserved crucifer blocks (U, F, and R) on 3 *Arabidopsis* chromosomes where 2 *CR* QTLs were detected in *A. thaliana*. These results suggest that some common ancestral genomic regions were involved in the evolution of *CR* genes in *B. rapa*.

## Introduction

*Plasmodiophora brassicae* Woronin, an obligate biotrophic protist, causes clubroot disease as a symptom of clubbed root in crucifers, such as *Brassica rapa*, *B. oleracea*, *B. napus*, *Raphanus sativus*, and *Arabidopsis thaliana* [1]. Limitations of cultural practices or chemical treatments for controlling clubroot disease have made breeding for clubroot-resistant cultivars desirable. However, the coexistence of multiple isolates in the field, broad genetic variation, and complex pathogenicity of *P. brassicae* [2–6] has made the breeding of resistant cultivars difficult, especially for cultivars having broad-spectrum or durable resistance. The most efficient way to breed such clubroot-resistant (CR) cultivars is to pyramid different *CR* genes.

Some valuable resistant sources to clubroot disease have been identified in *B. rapa*, especially in European fodder turnip (*B. rapa* ssp. *rapifera*) [1,7–9] and other *Brassica* crops, including *B. oleracea* and *B. napus*. Thus, the European Clubroot Differential (ECD) hosts 01–04 (*B. rapa* spp. *rapifera*) have been widely used as resistant donors in breeding CR cultivars in *Brassica* crops [4,10]. Genetic analysis indicated that resistance was controlled either by qualitative conferring of resistance by a single resistance gene in *B. rapa* or by quantitative conferring of resistance under polygene or quantitative trait loci (QTLs) in *B. oleracea* and *B. napus* [11]. In *B. napus*, Werner et al. [10] detected 19 *CR* QTLs on 8 chromosomes of *B. napus*, and some other major and minor genes were also demonstrated [2,12]. In *B. oleracea*, several *CR* QTLs were also indentified [13–16].In *A. thaliana*, one dominant *CR* gene were identified on chromosome 1 [17,18], several *CR* QTLs were detected on chromosomes 1, 4, and 5 [19]. QTLs involved in CR were also demonstrated in *B. rapa* [20,21]. 

Recent studies have revealed 8 *CR* genes positioned on 5 different chromosomes in *B. rapa*. With the exception of *Crr4* on chromosome A06 [21], which exhibited a minor only effects on resistance, the rest of the genes behaved as major genes, including *CRa* (on chromosome A03) [22], *CRb* (A03) [23], *Crr3* (A03) [24], *Crr1* (A08) and *Crr2* (A01) [20], *CRc* (A02), and *CRk* (A03) [25]. Recently, *Crr1* and *CRa* have been cloned and confirmed to carry Toll-Interleukin-1 receptor / nucleotide-binding site / leucine-rich repeat (TIR-NBS-LRR) structure [26,27]. However, all of the above-reported *CR* genes in *B. rapa* were identified using clubroot-resistant resources either from Chinese cabbage or from double haploid lines derived from a cross between Chinese cabbage and turnips [11]. As such, this raised the possibility that, besides previously identified *CR* genes, some *CR* genes may have been lost during the process of transferring *CR* genes from *CR* turnips into Chinese cabbage, since turnips have shown resistance to more isolates of *P. brassicae* than most of the commercialized resistance cultivars [5,28]. This is further supported by the fact that 10 *CR* QTLs are present in the A genome of *B. napus*, which was resynthesized by a cross between ‘Böhmerwaldkohl’ (*B. oleracea*) and ECD04 [10].

The interaction between *CR* genes and *P. brassicae* has been found to be isolate-specific and to confer broad-spectrum resistance in *B. oleracea* [13–15]. Werner et al. [10] detected 19 QTLs on 8 chromosomes in *B. napus*, and all of these QTLs were isolate-specific with respect to resistance. However, the effectiveness of *CR* genes has not been tested against more different pathotypes of *P. brassicae*, and isolate-specific resistance also has not been previously reported in *B. rapa*. For example, the pathotypes of races 2, 4, and another unknown race were used to identify *CR* genes. The pathotypes of these isolates were characterized by the Williams’ classification [29]. Among 8 *CR* genes, CRa, CRb, *CRk*, *Crr1*, *Crr3*, and *Crr4* were resistance to race 2 [21,23,25,30], and *CRk* and *CRc* exhibited resistance to an uncharacterized race [25]. In addition, *Crr1* and *Crr2* appeared to be resistant to race 4 in a complementary manner [20]. Indeed, there are more pathotypes of *P. brassicae* that exhibit significant differences in pathogenicity [7,29]. In addition, genetic changes in pathogen populations caused the erosion of commercial CR-resistant cultivars of Chinese cabbage (*B. rapa* spp. *pekenensis*), which were developed by the introduction of monogenes or oligogenes [5]. Hence, understanding the interactions between *CR* genes and *P. brassicae*, and the molecular mechanisms involved in CR could be an efficient strategy to control clubroot disease by breeding programs. 

The objective of this study was to (1) integrate unigene-derived microsatellite (UGMS) markers into a genetic linkage map of *B. rapa*, (2) identify molecular markers linked to novel *CR* genes in *B. rapa*, (3) explore the effects and specificities of *CR* genes involved in the control of 4 different *P. brassicae* isolates, and (4) compare the published *CR* genes with *CR* QTLs identified in this study and synteny regions where *CR* genes are located in between *B. rapa* and *A. thaliana*. 

## Materials and Methods

### Ethics statement

Four field isolates of *Plasmodiphora brassicae*, including Pb2, Pb4, Pb7, and Pb10, respectively, were provided by the following persons:

1. Prof. Xiangqun Shen at Shenyang Agricultural University, Liaoning;
2. Yuntian Bian, a farmer in Jilin;
3. Dr. Zhizhong Zhao at Shandong Academy of Agricultural Sciences in Shandong;
4. Prof. Ren Huang at Sichuan Agricultural University in Sichuan.

All of the field studies were carried out in a closed and protected green house in Shenyang Agricultural University. Therefore, the field studies did not involve endangered or protected species.

### Plant materials

Two subspecies of *B. rapa*, a Chinese cabbage (*B. rapa* ssp. *pekenensis*) inbred line C59-1 and turnip (*B. rapa* ssp. *repifera*) inbred line ECD04, were used as recurrent and donor parents, respectively. C59-1 is susceptible to clubroot disease, while the homogeneous line ECD04, which was isolated from ECD hosts and purified by self-crossing, is resistant to clubroot disease [7]. A population of 115 BC_1_F_1_ individuals was obtained by crossing the C59-1 line onto a single F_1_ plant, and this population was used to construct a genetic linkage map. For evaluation of the responses to field isolates of *P. brassicae*, young seedlings derived from seeds of each BC_1_F_1_ plant by self-pollination were used for inoculation of different field isolates under controlled conditions, respectively.

### Pathogen isolates

Four field isolates of *P. brassicae*, including Pb2, Pb4, Pb7, and Pb10, were collected from infected Chinese cabbage plants cultivated in 4 different areas of China: Liaoning, Jilin, Shandong, and Sichuan. Based on the Williams’ classification [29], Pb2, Pb4, Pb7, and Pb10 were characterized as pathotype races 2, 4, 7, and 10, respectively. After propagation on the susceptible Chinese cabbage, roots infected by each of the 4 different isolates were stored at -20°C until use. Resting spores were diluted to a density of 10^7^ spores per mL in sterile distilled water after isolation from homogenized clubbed roots. 

### Clubroot disease resistance test

To determine the genotype of each BC_1_F_1_, the seeds from the corresponding BC_1_F_2_ families were planted in 50-well multipots and maintained in a greenhouse under a 16-h photoperiod at 20–25°C. Resistance tests were performed in a randomized block design with 2 replications. Eighty-six BC_1_F_2_ families were tested against isolate Pb2, 84 were tested against Pb4, 88 were tested against Pb7, and 90 were tested against Pb10. For each isolate, 12 (one block) plants per BC_1_F_2_ family were tested. Twelve plants from each of two parental lines and F_1_ progeny were also included in all replicates, and were randomly placed between the randomized BC_1_F_2_ families. One-week-old seedlings were inoculated by application of 1 mL of resting spore suspension at the bottom of the stem base of each seedling. Six weeks after inoculation, symptoms of disease were scored as follows: 0 = no visible clubs, 1 = clubs usually confined to lateral roots, 2 = very slight clubs on main roots, 3 = moderate clubbing on main roots, 4 = larger clubs in main roots and slight clubs on lateral roots, and 5 = severe clubbing on main roots and lateral roots. The disease index (DI) was calculated according to the formula: DI = [(*n*_*1*_ + 2*n*_*2*_ + … + 5*n*_*5*_)/*N*_*T*_ × 5] ×100, where *n*_*i*_ is the number of plants with the symptom of *i* and *N*_*T*_ is the total number of plants tested. The DI for each BC_1_F_1_ individual was calculated from the mean grades of 2 replicates. 

### DNA extraction and marker analysis

DNA was extracted from the young leaves of 115 BC_1_F_1_ plants and parental lines according to the cetyl-trimethyl-ammonium-bromide method [31] with minor modifications. A total of 1099 SSRs, including 380 BAC-derived SSRs (designated by ‘cnu’, ‘nia’, and BRPGM) from Choi et al. [32], Kim et al. [33], and Li et al. [34], 74 genomic sequence-derived SSRs (prefixed by ‘hri’) from Suwabe et al. [21,35] and Choi et al. [32], 53 SSRs (prefixed by ‘pbc’, ‘Ra’, ‘aaf’, and ‘BnGMS’) from Choi et al. [32], 592 EST-derived SSRs, including 570 UGMSs developed in our laboratory (prefixed by ‘sau_um) [36] and 22 (prefixed by ‘ACMP’) from Ramchiary et al. [37], and 24 intron polymorphic (IP) markers from Panjabi et al. [38] were used for a polymorphism survey between the parental lines C59-1 and ECD04. 

To identify the location of the *B. rapa CR* genes in the genetic map constructed in this study, 22 previously reported markers linked to different *CR* genes [20,21,23,25,39,40] were also used. Procedures for the PCR assay and marker genotyping were conducted as described in the above reports. 

PCR products were resolved by electrophoresis on 8% polyacrylamide gels as described by Ge et al. [36]. Segregation of each marker in the BC_1_F_1_ population was visually scored.

### Construction of linkage map

The genetic map was constructed using JoinMap version 4 [41,42]. Logarithm of the odds (LOD) scores 4.0 to 6.0 was used to assign the markers into linkage groups (LGs) and Kosambi’s [43] mapping function was used to convert the recombination value into the map distance (cntiMorgans, cM). The threshold for goodness-of-fit was set to ≤ 5.0, with a recombination frequency of <0.4 and minimum LOD scores of 2.0. The map was drawn using Mapchart 2.1 [44].

### Statistical analysis and QTL mapping

Microsoft Excel was used to analyze the frequency distribution of mapping populations and their parents for resistance to each isolate of *P. brassicae*, using the DI of clubbed plants. Correlation analysis was conducted with SPSS software (SPSS, Inc., Chicago, IL, USA). 

QTL detection was performed using the composite interval mapping (CIM) function provided in Windows QTL Cartographer version 2.5 [45]. Tests for the presence of QTL were performed at 2 cM intervals using a 10 cM window and 5 background cofactors (Model 6). For declaring the presence of a QTL, genome-wide threshold values (*P* = 0.05) were estimated from 1,000 permutations of trait data across all genetic intervals [46,47]. The QTL locations were defined by the significance threshold of LOD value (3.3, 3.2, 3.8, and 3.0 for Pb2, Pb4, Pb7, and Pb10, respectively). QTLs were designated as *Pb* (for *Plasmodiophora brassicae*), followed *Ba* (for *Brassica rapa*), linkage group number, and QTL number. QTLs detected in overlapped confidence intervals were considered the same QTL region. Graphic representations of maps were generated using Mapchart 2.1 [44].

### Comparative analysis of clubroot resistance in *B. rapa* and *A. thaliana*

To find the physical locations of each *CR* QTL region identified in the present study and previous reports, the sequences of flanked markers were assigned to the *B. rapa* genome (http://www.brassicadb.org) by BLASTn. The sequences of each marker linked to *CR* loci were found by aligning the primer sequences to the *B. rapa* genome. When the primer pair was identical to the sequence fragment of the same chromosome and its defined length was similar to the respective marker, the sequences were considered to be the marker sequence. Additionally, the marker sequence was aligned against the genome sequences of *Arabidopsis* by BLASTn in TAIR (http://www.arabidopsis.org) and crucifer building blocks [48] in order to identify the syntenic regions between *B. rapa* and *A. thaliana*. Based on the *E* value ≤ 10^-10^, the syntenic region was determined if 2 or more common homologous loci existed in the corresponding regions between *B. rapa* and *A. thaliana*. When the *E*-value was between 10^-10^ and 10^-5^, they were also considered as homologous synteny region if the presence of SSR sequence was manually confirmed and neighboring SSR loci were relatively conserved. Markers showing homology to the *Arabidopsis* genome sequence or genes were mapped based on the physical positions of these genes.

## Results

### Linkage map with newly integrated UGMS

**Figure 1:**
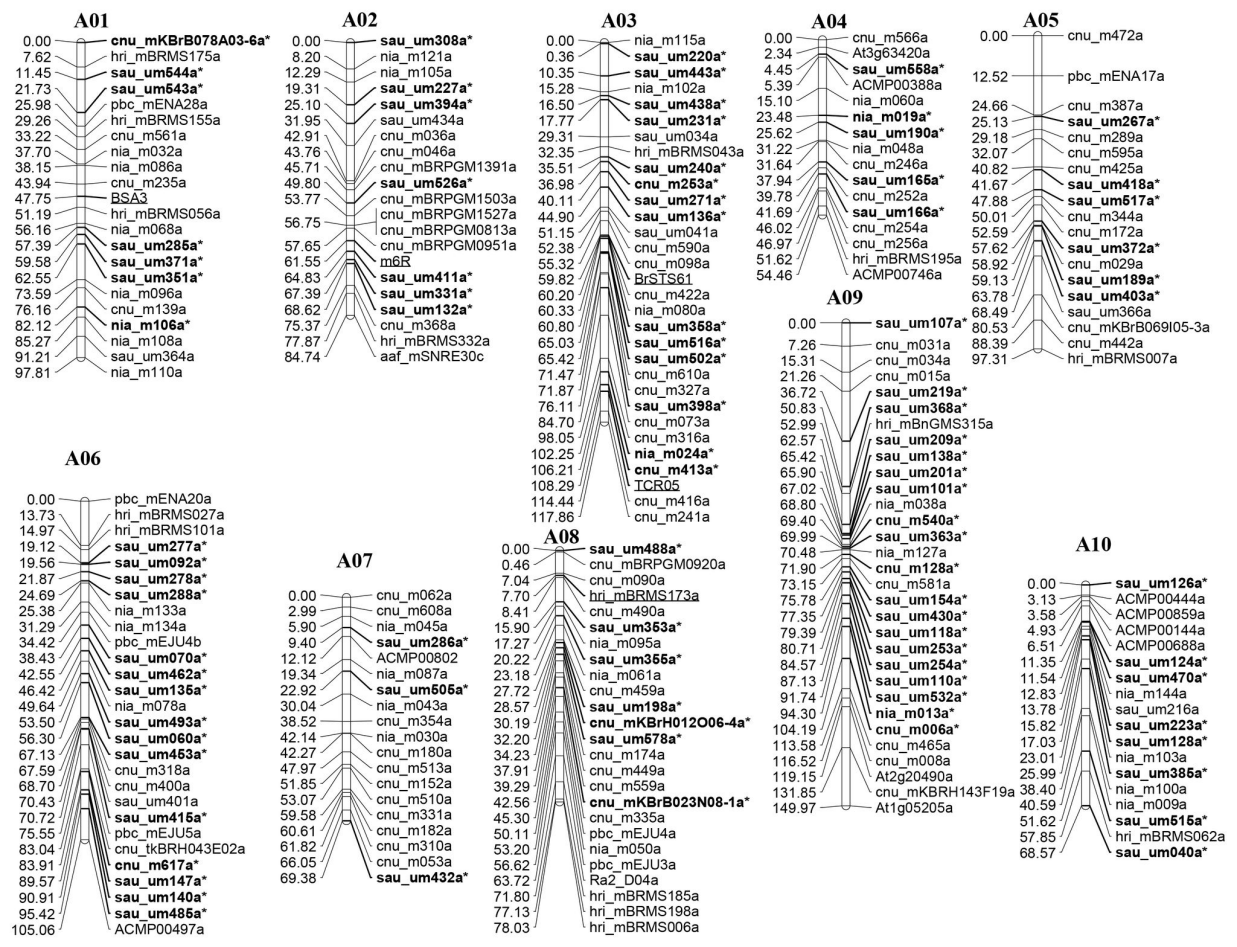
Genetic linkage map of *Brassica rapa*. Linkage groups were numbered A1 to A10 according to the anchor markers. The distances in centiMorgan were given on the left and the marker numbers are indicated on the right. The newly mapped markers were marked with boldface and asterisks. The markers linked to published clubroot resistance genes were underlined.

For the construction of the genetic linkage map, SSRs and *CR* gene-linked markers were screened for polymorphism between the parental lines. Of the 380 BAC-derived SSRs, 74 hri_BRMSs, and 53 markers prefixed by ‘pbc’, ‘Ra’, ‘aaf’, and ‘BnGMS’ screened, only 130 BAC-derived SSRs, 19 hri_BRMSs, and 18 other markers were polymorphic between ECD04 and C59-1. Among 560 successful amplifications from 592 EST-derived SSR markers, only 117 (20.9%) were polymorphic between the 2 parental lines. In addition, 5 IP markers displayed polymorphism. Of the 22 markers linked to 8 *CR* genes, only 5 were found to be polymorphic between the 2 parental lines. Thus, a total of 294 markers could be used to genotype the population. After excluding the seriously distorted and ungrouped markers, a total of 230 markers, including 132 genome sequence-derived SSRs, 93 UGMS SSRs, 5 markers linked to 5 *CR* genes, were assigned to the 10 linkage groups, corresponding to the 10 chromosomes of *B. rapa* (Figure 1). Alignment of marker sequence to the corresponding chromosome indicated that most of markers arrange in that order of physical position in the *B. rapa* genome (data not show).

Of the 93 UGMS SSRs, 78 UGMSs were newly mapped and integrated into the 10 linkage groups in this study, although they were previously developed by Ge et al. [36]. These UGMSs were distributed in all of the linkage groups, and the number of markers ranged from 3 in A07 to 15 in A09 (Table S1). Additionally, 13 BAC-derived SSRs were newly mapped. The total length of the map was 923.3 cM, with an average marker interval of 4.01 cM. The length of the individual linkage groups ranged from 54.5 cM in A04 to 150.0 cM in A09. Each linkage group was named according to the internationally accepted *Brassica* reference map based on the anchor SSR markers reported by Choi et al. [32], Kim et al. [33], Ge et al. [36], and Ramchiary et al. [37]. 

### Resistance to *P. brassicae* isolates

**Figure 2:**
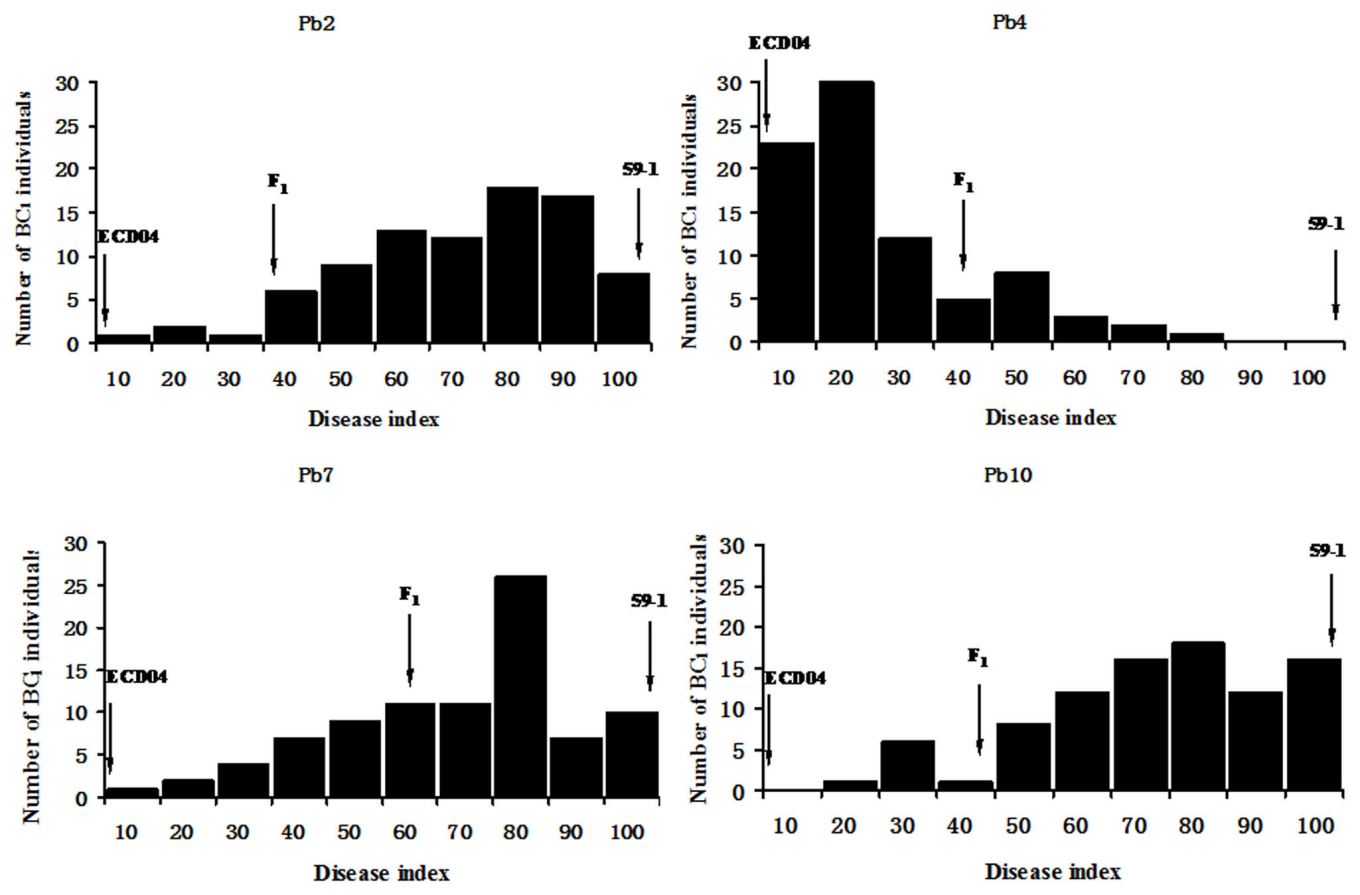
Frequency distributions of the disease index for clubroot resistance to the 4 isolates of *Plasmordium brassicae* in BC_1_F_2_ families.

Between 84 and 90 BC_1_F_2_ families from the BC_1_F_1_ population were tested with 4 pathotypes of *P. brassicae*. In each test, two parents and F_1_ were also included. The resistant parent ECD04 had a DI of 0.0, the susceptible parent C59-1 showed a DI of 100.0 (Figure 2). However, F_1_ plants showed an intermediate DI value between two parental lines. The frequency distribution of BC_1_F_2_ families for resistance to all isolates showed continuous segregation patterns (Figure 2). More individuals were closer to the susceptible parent C59-1 when inoculated with isolates Pb2, Pb7, and Pb10, while a large number of individuals were closer to ECD04 when isolate Pb4 was tested. These observations suggested that resistance to Pb4 is controlled by few genes with large genetic effects, while multiple genes are involved in resistance to the rest of the isolates. DI appeared different among the 4 isolates in the population, suggesting variation in the virulence of the 4 isolates. Meanwhile, the disease reaction was significantly correlated among the 4 isolates (Table 1). The lower correlation was found between isolate Pb4 with other isolates, indicating that different genes might control these different pathotypes. However, a high correlation was observed between Pb2 and Pb7. 

**Table 1:** Correlation coefficients among the disease index values after inoculation of BC_2_F_2_ families derived from the cross C59-1 × ECD04 with 4 isolates of *Plasmordium brassicae*.

	Pb4	Pb7	Pb10
Pb2	0.454**	0.721**	0.565**
Pb4	–	0.407**	0.401**
Pb7	–	–	0.550**

^**^ Significant at *P* < 0.01

### Isolate-specific QTLs for CR in *B. rapa*

**Figure 3:**
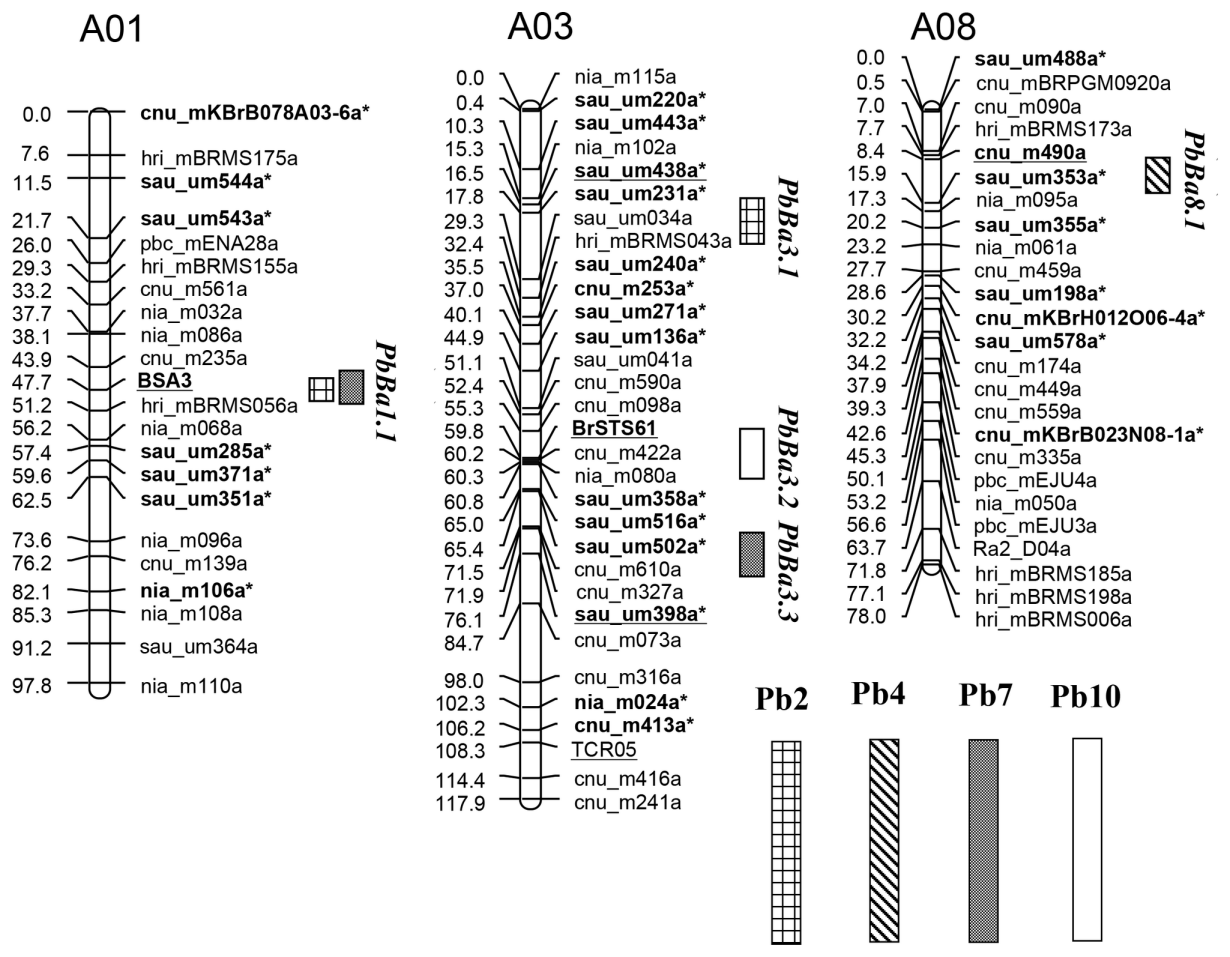
Linkage maps of four *Brassica rapa* chromosomes with detected QTL for clubroot resistance. The distances in centiMorgan are given on the left and the marker numbers are indicated on the right. The markers on the peak of each QTL are underlined and marked with boldface. The boxes indicate confidence intervals of QTL detected with the 4 isolates.

Composite interval mapping identified 6 QTLs for partial resistance against 4 *P. brassicae* isolates, which were positioned in 5 *CR* QTL regions (Figure 3, Table 2). The resistance alleles of all QTLs were found to be contributed by the resistant parent ECD04. The phenotypic variance explained by each QTL ranged from 12.2% to 35.2% depending on the respective isolate. The large range of phenotypic variance could be explained by different disease pressures on the isolates.

**Table 2:** Details of the quantitative trait loci detected for clubroot resistance against 4 different isolates of *Plasmordium brassicae*.

Isolate	Linkage group	QTL name	LOD**^a^**	Closest marker	Peak position (cM)	Confidence interval (cM)	*R* ^2^(%)**^b^**	A^c^
Pb2	A01	*PbBa1.1*	4.5	BSA3	47.8	46.0–49.9	13.2	+0.72
	A03	*PbBa3.1*	4.1	sau_um438a	16.5	15.3–23.2	12.2	+0.71
Pb4	A08	*PbBa8.1*	8.5	cnu_m490a	10.4	8.4–14.6	35.2	+1.10
Pb7	A01	*PbBa1.1*	5.0	BSA3	46.0	44.0–49.8	18.7	+0.95
	A03	*PbBa3.3*	5.2	sau_um398a	76.1	72.5–79.9	16.5	+0.89
Pb10	A03	*PbBa3.2*	4.5	BrSTS61	60.3	54.8–63.2	14.0	+0.79

^a^ The logarithm of odds (LOD). LOD indicates the likelihood at the peak of the QTL.

^b^ The *R* ^*2*^ indicates the percentage of phenotypic variance explained by each QTL.

^c^ Additive effect value of the QTL. Positive additivity indicates that the QTL allele originating from the parental ECD04 was resistant to clubroot disease.

For isolate Pb2, 2 QTLs showing partial resistance to clubroot disease were distributed on A01 and A03. The first QTL, designated as *PbBa1.1*, was linked to the marker BSA3 (LOD = 4.5) on A01. The other QTL (*PbBa3.1*) was linked to the makers sau_um438a (LOD = 4.1) on A03. One possible QTL (*PbBa3.3*) was also detected near the marker cnu_m327 (LOD=2.8) on A03 (Table S3). Since *PbBa3.3* was identified as a major QTL by Pb7, it might be a positive QTL for controlling partial resistance to Pb2. *PbBa3.1* and *PbBa3.3* were independent since they were mapped to the different regions of A03. For isolate Pb4, only *PbBa8.1* was detected near the marker cnu_m490a (LOD = 8.5) on A08. 

For isolate Pb7, 2 QTLs showed linkage to the BSA3 marker (LOD = 5.0) on A01 and to sau_um398a (LOD = 5.2) on A03. Of the two QTLs, one was located in the region of *PbBa1.1*, one was a newly identified QTL. This new QTL was designated as *PbBa3.3*. In addition, one positive QTL (LOD=2.9) was identified in the region of *PbBa8.1* although it did not show significance at the LOD threshold of 3.8 (Table S3). In this interval, where previous identified *Crr1* was located [20], a major QTL *PbBa8.1* was also detected by Pb4. For isolate Pb10, only one QTL *PbBa3.2* was identified by linkage to the BrSTS61 markers (LOD = 4.5) on A03. This QTL was independent of all QTLs detected from isolates Pb2, Pb4, and Pb7. 

Among the QTLs revealed in this study by the 4 different isolates, only 1 single locus of *PbBa8.1* was involved in controlling resistance against the isolate Pb4, and also *PbBa3.2* to Pb10. Resistance to the remaining 2 isolates was controlled by the polygenes, indicating a quantitative effect against the different isolates in line ECD04. In addition, 3 *CR* QTL regions contributed partial resistance to 2 different isolates when *PbBa3.3* and *PbBa8.1* detected by Pb2 and Pb7, respectively, were considered to be positive. For instance, 2 QTLs, *PbBa1.1* on chromosome A01 in the region between cnu_m235a and hri_mBRMS056 and *PbBa3.3* on A03 in the region between cnu_m327a and cnu_m073a, provide partial resistance to both Pb2 and Pb7, explaining 7.8%–18.7% of the phenotypic variation (Table S3). *PbBa8.1* in the region between hri_mBRMS173 and sau_um353a was partial resistance to Pb4 and Pb7. Another 2 QTLs were found to contribute partial resistance only to 1 isolate, i.e., *PbBa3.1* was partial resistance to isolate Pb2, while *PbBa3.2* was partial resistance to Pb10. These 2 QTLs explained 12.2% and 14.0% of the phenotypic variation, respectively. The results obtained here suggested the presence of isolate-specific-resistant QTLs to clubroot disease in *B. rapa*.

### Syntenic analysis of *CR* QTL regions in *B. rapa* and *A. thaliana*

**Figure 4:**
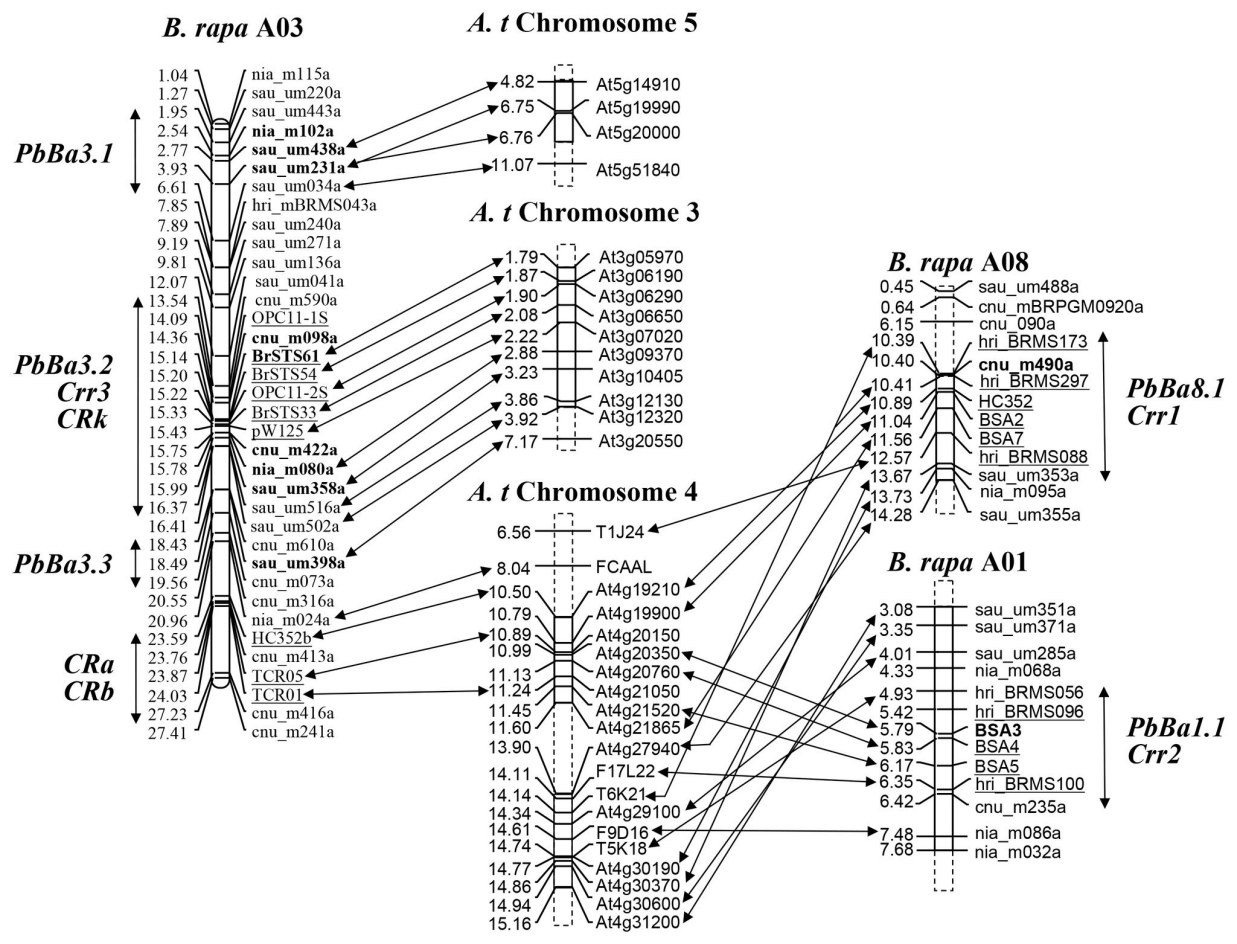
Microsynteny of QTL regions for clubroot resistance between *Brassica rapa* and *Arabidopsis thaliana*. The number on the left of vertical bars indicates the physical position in megabase (Mb) of chromosomes either from *B. rapa* or *Arabidopsis*. The clubroot resistance (CR) QTL regions are indicated by vertical lines with 2 arrows. The markers linked to *CR* QTLs in each linkage map are indicated in boldface. The markers linked to each published *CR* locus are underlined. The *Arabidopsis* genes or genomic regions (bacterial artificial chromosome clones) corresponding to the markers on the linkage groups of *B. rapa* are connected by lines with 2 arrows.

Published available markers, especially those markers closely linked to the previously mapped major *CR* genes, allowed us to compare the identical QTLs revealed in this study to those previously reported *CR* genes. Meanwhile, it was also possible to identify other *CR* genes that may have been lost during introgression of *CR* genes from *CR* turnip into Chinese cabbage. Of 22 *CR* gene-linked markers, 5 markers, including BSA3, m6R, BrSTS061, TCR05, and BRMS173, which are closely linked to *Crr2*, *CRc*, *Crr3*, *CRb*, and *Crr1*, respectively, showed polymorphism between the two parental lines ECD04 and C59-1. Additionally, all the sequences of markers linked to those *CR* loci were aligned to the corresponding chromosome and could arrange in that order of physical position in the *B. rapa* genome (Figure 4). *PbBa1.1* and *Crr2* were mapped to the region from 4.93 to 6.42 Mb on chromosome A01, and *PbBa8.1* and *Crr1* were in the region from 10.39 to 13.67 Mb on A08. Four *CR* QTLs were positioned on A03, including *CRa* and *CRb* in the region of 23.59–27.23 Mb, *PbBa3.1* in the region of 1.95–6.61 Mb, *PbBa3.2*, *CRk*, and *Crr3* in the region of 13.54–16.37 Mb, and *PbBa3.3* in the region of 18.43–19.56 Mb. These results suggested that *PbBa1.1*, *PbBa3.2*, and *PbBa8.1* was identical or closely linked to *Crr2*, *CRk* and *Crr3*, and *Crr1*, respectively. 

However, there were still 2 *CR* loci identified in this study for which we could not find resistant counterparts that were previously reported in the A genome, including *PbBa3.1* and *PbBa3.3*, at the different physical regions on the same chromosome A03. Interestingly, no QTLs were detected in the regions where the common linkage markers of m6R and TCR05 were located, implying that homologous genes to *CRc* on A02 and *CRb* on A03 did not exist in either parental line in this study. 

Additionally, DNA sequences of the markers linked to each *CR* gene were also compared with the genomic sequences of *Arabidopsis* to find syntenic regions between *B. rapa* and *A. thaliana*. It was revealed that each *CR* QTL region could be assigned to a syntenic region in *Arabidopsis* chromosomes (Figure 4; Table S2). *PbBa1.1* and *Crr2* from A01, *PbBa8.1* and *Crr1* from A08, and *CRa* and *CRb* from A03 were aligned to the U block one single genomic region from 8.04 to 15.16 Mb on *Arabidopsis* chromosome 4. The markers in the region of *PbBa3.2*, *CRk*, and *Crr3* showed colinearity to the F block on *Arabidopsis* chromosome 3 as well. In addition, *PbBa3.3* aligned to the F block as *PbBa3.2*. The syntenic region of *PbBa3.1* was found in the R block on chromosomes 5 of *Arabidopsis*.

## Discussion

### Mapping of UGMS markers in *B. rapa*

UGMS markers have great advantages and utilities for molecular breeding and evolutionary studies since they are developed from coding regions and show high transferability across species. Although the development and mapping of UGMS markers in *B. rapa* have advanced further in recent years [36,37,49], the exact genomic locations of many UGMS markers have not yet been identified. Here, we assigned 93 UGMS markers, of which 78 were newly mapped into 10 linkage groups. UGMS markers showed much higher polymorphism (20.9%) than the 14% previously reported by Ramchiary et al. [37] and the 16.4% previously reported by Ge et al. [36]. This can be explained by the different parental lines used in each study. Ramchiary et al. [37] and Ge et al. [36] used 2 diverse Chinese cabbage lines (*B. rapa* ssp. *pekinensis*). In this study, we used 2 different subspecies of *B. rapa*, Chinese cabbage and turnip (*B. rapa* ssp. *repifera*). Greater polymorphism (20%) was also observed between Chinese cabbage and rapid-cycling *B. rapa* [37]. These findings indicated that SSRs located in coding regions were conserved, but exhibited more variance between subspecies of *B. rapa*. 

### *CR* genes in the A genome of *Brassica* species

In total, 5 *CR* QTL regions originating from ECD04 were identified from 4 different isolates of *P. brassicae* and were spread over 3 chromosomes of *B. rapa*. Comparative results indicated that ECD04 possessed the homologous or identical *CR* alleles *Crr1*, *Crr2*, and *Crr3*, which have previously been reported [20,24]. Meanwhile, ECD04 was also found to contain 2 additional novel loci, including *PbBa3.1* and *PbBa3.3* on A03. However, all QTLs explained from 12.4% to 35.2% of phenotypic variance, indicating that not all of the genetic variance was explained by these QTLs. Further, only one QTL was detected with the isolate Pb10. This may result from the backcross population and its relative smaller population size or from the choice of the significance threshold, which may have prevented the detection of minor QTLs. For example, *PbBa3.3* and *PbBa8.1* were detected by Pb2 and Pb7 at the respective LOD value of 2.8 and 2.9, but not at the LOD significance threshold value of 3.3 and 3.8. However, these 2 QTLs were confirmed to be major QTLs for partial resistance to the isolate of Pb7 and Pb4, respectively. Therefore, *PbBa3.3* and *PbBa8.1* might be the positive QTLs, and act with minor effects to Pb2 and Pb7, respectively. This was also supported by the evidence that the genomic region of *PbBa8.1* was aligned to the U block where a *CR* QTL (*Pb-At4*) was located in *Arabidopsis* [19]. The effects of these 2 QTLs remain to be confirmed in the later study. Phenotypic variance in the range of 12.4% to 67.5% explained by the respective *CR* QTLs was also observed by Werner et al. [10], who used ECD04 as a resistant donor parent in the resynthesized *B. napus*.

Previously mapped *CR* genes, such as CRa, CRb, *CRk*, and *Crr3*, and also *PbBa3.1*, *PbBa3.2*, and *PbBa3.3* identified in this study, were all distributed on chromosome A03. These *CR* loci were either independent or located in the near region. Physical mapping of the public markers linking to *CRa* and *CRb* revealed that they were located between 23.59 and 27.23 Mb. Cloning of *CRa* have confirmed that it is positioned in this region [27]. The *CRb* gene remains to be cloned for understanding the relationship between *CRa* and *CRb*. However, we did not detect any counterpart QTLs on this region, although TCR05 linked to *CRb* was mapped. Sakomoto et al. [25] suggested that *CRk* was next to *Crr3*. We also found that the *PbBa3.2*, *Crr3*, and *CRk* were located in the physical region from 13.54 to 16.37 Mb, indicating that they were the same allele or closely linked. However, the physical location of the major QTL *PbBa3.1* (LOD = 4.1) between 1.95 and 6.61 Mb and another major QTL *PbBa3.3* (LOD = 5.2) between 18.43 and 19.56 Mb suggested that they were independent from other *CR* genes on A03. To reveal the precise relationship between these *CR* loci, fine mapping or even cloning of these QTLs is required in future experiments. 

Combinations of 2 or 3 *CR* genes were suggested in the *B. rapa* ECD hosts ECD01 to ECD04 [50,51]. However, resistant sources were not found in the germplasm of Chinese cabbage [4], the main leafy vegetable crops in East Asian countries. Therefore, the CR European turnips have been used to breed CR cultivars of Chinese cabbages by introducing respective *CR* genes, such as *CRc* and *CRk* from ECD01, *CRa* and *CRb* from ECD02, *Crr1* and *Crr2* from Siloga, and *Crr3* from Milan White [52]. However, CR cultivars have been challenged from clubroot disease, while CR turnips are still resistant to clubroot [5,28]. This suggests that some of *CR* genes were lost during introgression of *CR* genes from *CR* turnips into Chinese cabbage. The QTLs identified here contribute only partial resistance. It is therefore easy to see why partial resistance is lost during the intrgression process as demonstrated by Cowling et al. [53]. The *Crr2* gene has been shown to be lost during breeding of commercial CR Chinese cabbage, but has also been found to be resistant to more virulent pathotypes in combination with *Crr1* [20]. It is also possible that more durable CR turnips were not used as a resistant resource. The identification of new *CR* QTLs indicates that some other *CR* genes are still present in CR turnips. The molecular markers linked to these partial resistance genes will be informative for the breeding of CR cultivars in *B. rapa* by pyramiding *CR* genes. 

### Isolate-specific resistance to *P. brassicae* in *Brassica* crops

The differential effects of published *CR* genes toward different pathotypes of *P. brassicae* indicated isolate-specific resistance in *B. rapa* [11]. QTL mapping of CR with 4 different *P. brassicae* isolates allowed us to further anatomize the performance or specificity of each *CR* gene identified in this study. QTL analysis indicated that none of QTLs identified here were effectively resistant to all isolates tested, they contribute only partial resistance. *PbBa3.1* and *PbBa3.2* showed partial resistance to only 1 isolate, Pb2 and Pb10, respectively. Others were effective against 2 different isolates. Resistance to more than 2 different isolates was also controlled by *CRb* [23] and *CRk* [25]. All these results suggest that *CR* is isolate-specific in *B. rapa*. Such isolate-specific partial resistance was also found in the resynthesized *B. napus* for which ECD04 was used as a resistant donor parent in a study by Werner et al. [10]. The interaction of these *CR* genes with other pathotypes remains to be further studied with more isolates. 

However, the CR mechanism through which *Crr2* acts as an enhancer for the expression of *Crr1*, facilitating resistance to more virulent pathogen infection, rather than isolate specificity, was also hypothesized by Suwabe et al. [21]. Similarly, *PbBa8.1* (LOD = 8.5), which colocalized with *Crr1*, was effectively resistant to the Pb4 isolate, but only slightly resistant (LOD = 2.9) to Pb7. The expression of *PbBa1.1* and *PbBa3.3*, conferring partial resistance to Pb7, might require the aid of *PbBa8.1*. *PbBa1.1* in the same genomic region as *Crr2* and the additional QTL *PbBa3.3* were partial resistant to Pb2 and Pb7 through different mechanisms. *PbBa1.1* acted as a major resistance gene against both isolates. In contrast, *PbBa3.3* acted as a major resistance gene against Pb7, but had a weaker effect toward Pb2. This suggested that the same *CR* locus in *B. rapa* can act as either a major gene or as a minor QTL, depending on the isolate. Supporting results have also been obtained in other *Brassica* crops. In *B. napus*, a dominant major gene for resistance to isolate Pb137-522 of *P. brassicae* had a weaker effect against K92-16 [2]. Rocherieux et al. [15] identified a major resistance gene that regulated resistance to isolates Pb137-522, K92, and K92-16, but showed weaker effects against Ms6 and eH in *B. oleracea*.

### The genetic origin and candidate loci for CR in *B. rapa*

Based on comparative mapping, the genomic regions containing *CR* genes identified in the present study and earlier studies in *B. rapa* were aligned to the 3 blocks (R, F, and U) on the 3 chromosomes of *A. thaliana*. Furthermore, these studies permitted a comparison between the *B. rapa CR* QTL and those *CR* QTLs identified in *Arabidopsis*. *PbBa3.2*, together with *Crr3* and *CRk*, revealed the conserved F block on the top chromosome 3 of *Arabidopsis*, as reported by Saito et al. [39]. We also found that *PbBa3.3*, a novel QTL, was aligned to the F block. Three genomic regions containing *CRa* and *CRb*, *PbBa1.1* and *Crr2*, and *PbBa8.1* and *Crr1* were mapped to the U block between 6.56 and 15.16 Mb of chromosome 4, where *Pb-At4*, a QTL for CR in *Arabidopsis*, is located [19]. The common origin of *Crr1* and *Crr2* was also presented by Suwabe et al. [21]. Three hypothetical resistance genes that have nucleotide-binding site and leucine-rich repeat (NBS-LRR) motifs are located in the *Pb-At4* region. Among them, *RPS2* confers specific resistance to *Pseudomonas syringae* pv. Tomato [54]. *PbBa3.1* also showed synteny to the *CR* QTL region of *Arabidopsis*. A major *CR* QTL, *Pb-At5.1*, corresponding to *PbBa3.1*, was identified in the R block on the chromosome 5 of *Arabidopsis* [19]. Identification of the syntenic regions about *CR* genes between *B. rapa* and *A. thaliana* indicates that several common ancestral genomic regions are possibly involved in the evolution of *CR* genes in *B. rapa*. For example, the common ancestor of *Pb-At4* on the U block might have diverged into triplicate resistance genes residing in the *B. rapa* chromosomes A01, A03, and A08 as revealed by Suwabe et al [21]. The evolutionary origin for QTLs controlling the same morphological traits was also found in the conserved U blocks of the *Brassica* genome [55]. . Without a doubt, these *CR* genes may also have evolutionally originated from clustering resistance genes, since the clustering of disease resistance genes is common in the *Arabidopsis* [56] and other plant genomes [57,58]. However, our data showed that 2 novel *CR* QTLs, *PbBa3.1* and *PbBa3.2*, were from the R and F block in the *Arabidopsis* chromosome 3 and 5, respectively. Identification of the R and F blocks in this study indicates that more than one ancestral gene were involved in the evolution of the *CR* genes in *B. rapa*. In addition, we also observed that *PbBa3.1*, *PbBa3.2* and *PbBa3.3* was only located on one paralogous block (R and F, respectively) even these 2 blocks are present in 3 chromosomes of *B. rapa* [48], suggesting that the loss of *CR* gene might be happened during the triplication event of the *B. rapa* genome, or due to the functional inactivity of paralogous gene. There is also one possibility that the isolates employed in this study limited to find the conserved R and F blocks in other chromosomes of *B. rapa* due to the presence of isolate-specific resistance to clubroot disease.

Identification of candidate loci in the *Arabidopsis CR* QTL region will be informative for the cloning of *CR* genes in *B. rapa*. However, some genes involved in the auxin response, signaling pathways, and cell division should be also considered to be candidates, since the symptoms of clubbed root are likely caused by abnormal cell enlargement and uncontrolled cell division [59]. Fine mapping and cloning of underling *CR* genes will provide a fundamental understanding of the mechanisms of CR and should help with the development of appropriate programs for breeding CR cultivars in *Brassica* crops.

## Supporting Information

Table S1 **Details of the *Brassica rapa* linkage map.**
(DOC)

Table S2 **Homologous of the marker sequences located in the clubroot resistance QTL regions between *Brassica rapa* and *Arabidopsis thaliana*.**
(DOC)

Table S3 **Details of 6 major and 2 positive QTLs for clubroot resistance in *Brassica rapa*.**
(DOC)
