# Supplementary material for: Identification of Novel QTLs for Isolate-Specific Partial Resistance to Plasmodiophora brassicae in Brassica rapa
Source: PLoS One. 2013 Dec 20;8(12):e85307. doi: 10.1371/journal.pone.0085307 (PMC3869933; doi:10.1371/journal.pone.0085307)
Supplement: Table S1 — Details of the Brassica rapa linkage map. (DOC) [file pone.0085307.s001.doc]

Title: Identification of novel QTLs for isolate-specific partial resistance to *Plasmodiophora brassicae* in *Brassica rapa*

**Journal name:** Plos one

**Author name:** Jingjing Chen, Jing Jing, Zhongxiang Zhan, Teng Zhang, Chunyu Zhang, Zhongyun Piao

**Corresponding author:**

Zhongyun Piao, College of Horticulture, Shenyang Agricultural University, Shenyang 110866, China. Tel: +86-24-88487143, Fax: +86-24-88487145, E-mail: zypiao@syau.edu.cn

Chunyu Zhang, National Key Laboratory of Crop Genetic Improvement and College of Plant Science and Technology, Huazhong Agricultural University, Wuhan 430070, China. Tel: +86-27-87287563, Fax: +86-27-87280016, E-mail: zhchy@mail.hzau.edu.cn

**Table S1. Details of the *Brassica rapa* linkage map**

| Linkage group | Number of different marker types | | | | Total length (cM) | Average distance (cM) |
| --- | --- | --- | --- | --- | --- | --- |
|  | Genome sequence-derived SSRs^a^ | UGMS^b^ | Public markers linked to CR genes | Total |  |  |
| A1 | 15 (2) ^c^ | 6 (5） | 1 | 22 | 97.8 | 4.45 |
| A2 | 12 | 8 (7） | 1 | 21 | 84.7 | 4.03 |
| A3 | 16 (3) | 13 (11） | 2 | 31 | 117.9 | 3.80 |
| A4 | 10 (1) | 6 (4） |  | 16 | 54.5 | 3.41 |
| A5 | 12 | 7 (6） |  | 19 | 97.3 | 5.12 |
| A6 | 12 (1) | 16 (14） |  | 28 | 105.1 | 3.75 |
| A7 | 15 | 4 (3) |  | 19 | 69.4 | 3.65 |
| A8 | 19 (2) | 5 (5） | 1 | 25 | 78.0 | 3.12 |
| A9 | 16 (4) | 15 (15) |  | 31 | 150.0 | 4.84 |
| A10 | 5 | 13 (8) |  | 18 | 68.6 | 3.81 |
| Total | 132 (13) | 93（78） | 5 | 230 | 923.3 | 4.01 |

^a^ Genome sequence-derived SSRs include ‘cnu’, ‘nia’, ‘hri’, ‘pbc’, and ‘BnGMS’ markers. In addition, 3 IP markers are included.

^b^ UGMS markers include ‘ACMP’ and ‘sau_um’ markers.

^c^ Numbers in parenthesis indicate that markers newly mapped in this study.
