# Supplementary material for: Identification of Novel QTLs for Isolate-Specific Partial Resistance to Plasmodiophora brassicae in Brassica rapa
Source: PLoS One. 2013 Dec 20;8(12):e85307. doi: 10.1371/journal.pone.0085307 (PMC3869933; doi:10.1371/journal.pone.0085307)
Supplement: Table S2 — Homologous of the marker sequences located in the clubroot resistance QTL regions between Brassica rapa and Arabidopsis thaliana. (DOC) [file pone.0085307.s002.doc]

Title: Identification of novel QTLs for isolate-specific partial resistance to *Plasmodiophora brassicae* in *Brassica rapa*

Journal name: Plos one

Author name: Jingjing Chen, Jing Jing, Zhongxiang Zhan, Teng Zhang, Chunyu Zhang, Zhongyun Piao

Corresponding author:

Zhongyun Piao, College of Horticulture, Shenyang Agricultural University, Shenyang 110866, China. Tel: +86-24-88487143, Fax: +86-24-88487145, E-mail: zypiao@syau.edu.cn

Chunyu Zhang, National Key Laboratory of Crop Genetic Improvement and College of Plant Science and Technology, Huazhong Agricultural University, Wuhan 430070, China. Tel: +86-27-87287563, Fax: +86-27-87280016, E-mail: zhchy@mail.hzau.edu.cn

**Table S2.** Homologous of the marker sequences located in the clubroot resistance QTL regions between *Brassica rapa* and *Arabidopsis thaliana*

| QTL region | Marker name^a^ | *A. thaliana* | | | |
| --- | --- | --- | --- | --- | --- |
|  |  | Chromosome | Gene model or BAC clone | Distance (kb) | E-value |
| *PbBa1.1* | nia_m086a | 4 | F9D16 | 14610 | 1.00E-35 |
|  | cnu_m235a |  |  |  |  |
|  | BSA3 | 4 | At4g20350 | 10988 | 0 |
|  | hri_mBRMS056 |  |  |  |  |
|  | nia_m068a |  |  |  |  |
|  | sau_um285 | 4 | At4g29100 | 14341 | 9.00E-09 |
| *PbBa3.1* | nia_m102a | 5 | At5g13740 |  |  |
|  | sau_um438 | 5 | At5g14910 | 4823 | 9.00E-18 |
|  | sau_um231 | 5 | At5g19990 | 6752 | 2.00E-25 |
|  |  |  | At5g20000 | 6756 | 2.00E-19 |
|  | sau_um034 | 5 | At5g51840 | 21072 | 4.00E-19 |
|  | sau_um240 | 2 | At2g33620 | 14234 | 8.00E-07 |
|  | sau_um271 | 2 | At2g38670 | 16168 | 3.00E-06 |
| *PbBa3.2* | cnu_m098a |  |  |  |  |
|  | BrSTS61 | 3 | At3g05970 | 1786 | 2.00E-22 |
|  | cnu_m422a |  |  |  |  |
|  | nia_m080a | 3 | At3g09370 | 2879 | 1.00E-09 |
|  | sau_um358 | 3 | At3g10405 | 3233 | 5.00E-08 |
|  | sau_um516 | 3 | At3g12130 | 3864 | 2.00E-08 |
|  | sau_um502 | 3 | At3g12320 | 3924 | 2.00E-21 |
| *PbBa3.3* | cnu_m610a | 3 | T32A11 |  | 3.00E-05 |
|  | sau_um398 | 3 | At3g20550 | 7174 | 6.00E-05 |
|  | cnu_m073a |  |  |  |  |
| *PbBa8.1* | cnu_090 | 1 | At1g80890 | 30397 | 2.00E-42 |
|  | hri_mBRMS173 | 4 | T6K21 | 14140 | 2.00E-22 |
|  | cnu_m490a |  |  |  |  |
|  | sau_um353 | 4 | At4g30370 | 14858 | 6.00E-06 |
|  | nia_m095a | 4 | At4g30190 | 14770 | 3.00E-06 |
|  | sau_um355 | 4 | At4g27940 | 13904 | 1.00E-09 |

^a^ Markers linked to *CR* QTLs were shaded.
