# Supplementary material for: Identification of Novel QTLs for Isolate-Specific Partial Resistance to Plasmodiophora brassicae in Brassica rapa
Source: PLoS One. 2013 Dec 20;8(12):e85307. doi: 10.1371/journal.pone.0085307 (PMC3869933; doi:10.1371/journal.pone.0085307)
Supplement: Table S3 — Details of 6 major and 2 positive QTLs for clubroot resistance in Brassica rapa. (DOC) [file pone.0085307.s003.doc]

Title: Identification of novel QTLs for isolate-specific partial resistance to Plasmodiophora brassicae in *Brassica rapa*

Journal name: Plos one

Author name: Jingjing Chen, Jing Jing, Zhongxiang Zhan, Teng Zhang, Chunyu Zhang, Zhongyun Piao

Corresponding author:

Zhongyun Piao, College of Horticulture, Shenyang Agricultural University, Shenyang 110866, China. Tel: +86-24-88487143, Fax: +86-24-88487145, E-mail: zypiao@syau.edu.cn

Chunyu Zhang, National Key Laboratory of Crop Genetic Improvement and College of Plant Science and Technology, Huazhong Agricultural University, Wuhan 430070, China. Tel: +86-27-87287563, Fax: +86-27-87280016, E-mail: zhchy@mail.hzau.edu.cn

**Table S3.** Details of 6 major and 2 positive QTLs for clubroot resistance in *Brassica rapa*

| Isolate | Linkage group | QTL name | LOD^a^ | Closest marker | Peak position (cM) | Confidence interval (cM) | *R*^2^(%)^b^ | A^c^ | Threshold values (LOD) | Significance |
| --- | --- | --- | --- | --- | --- | --- | --- | --- | --- | --- |
| Pb2 | A01 | *PbBa1.1* | 4.5 | BSA3 | 47.8 | 46.0–49.9 | 13.2 | +0.72 | 3.3 | Yes |
|  | A03 | *PbBa3.1* | 4.1 | sau_um438a | 16.5 | 15.3–23.2 | 12.2 | +0.71 | 3.3 | Yes |
|  | A03 | *PbBa3.3* | 2.8 | cnu_m327a | 71.9 | 71.5–83.1 | 7.8 | +0.57 | 3.3 | No |
| Pb4 | A08 | *PbBa8.1* | 8.5 | cnu_m490a | 10.4 | 8.4–14.6 | 35.2 | +1.10 | 3.2 | Yes |
| Pb7 | A01 | *PbBa1.1* | 5.0 | BSA3 | 46.0 | 44.0–49.8 | 18.7 | +0.95 | 3.8 | Yes |
|  | A03 | *PbBa3.3* | 5.2 | sau_um398a | 76.1 | 72.5–79.9 | 16.5 | +0.89 | 3.8 | Yes |
|  | A08 | *PbBa8.1* | 2.9 | cnu_m490a | 10.4 | 9.8–15.9 | 9.2 | +0.71 | 3.8 | No |
| Pb10 | A03 | *PbBa3.2* | 4.5 | BrSTS61 | 60.3 | 54.8–63.2 | 14.0 | +0.79 | 3.0 | Yes |

^a^The logarithm of odds (LOD). LOD indicates the likelihood at the peak of the QTL.

^b^The *R^2^* indicates the percentage of phenotypic variance explained by each QTL.

^c^Additive effect value of the QTL. Positive additivity indicates that the QTL allele originating from the parental ECD04 was resistant to clubroot disease.
